# Antimicrobial susceptibility and molecular epidemiology of extended-spectrum beta-lactamase-producing Enterobacteriaceae from intensive care units at Hamad Medical Corporation, Qatar

**DOI:** 10.1186/s13756-016-0103-x

**Published:** 2016-02-09

**Authors:** Mazen A Sid Ahmed, Devendra Bansal, Anushree Acharya, Asha A. Elmi, Jemal M Hamid, Abuelhassan M Sid Ahmed, Prem Chandra, Emad Ibrahim, Ali A Sultan, Sanjay Doiphode, Naser Eldin Bilal, Anand Deshmukh

**Affiliations:** Division of Clinical Microbiology, Department of Laboratory Medicine and Pathology, Microbiology Section, Hamad Medical Corporation, Doha, Qatar; Department of Microbiology and Immunology, Weill Cornell Medicine-Qatar, Doha, Qatar; College of Medical Laboratory Sciences, University of Khartoum, Khartoum, Sudan

**Keywords:** Gram-negative bacteria, Extended-spectrum beta-lactamase, Antimicrobial Susceptibility, Molecular epidemiology, Qatar

## Abstract

**Background:**

The emergence of extended-spectrum beta-lactamase (ESBL)-producing isolates has important clinical and therapeutic implications. High prevalence of ESBL-producing Enterobacteriaceae has been reported in the literature for clinical samples from a variety of infection sites. The present study was undertaken to evaluate the prevalence of ESBL-producing Enterobacteriaceae, and to perform molecular characterization and antimicrobial susceptibility testing of clinical isolates from patients admitted to the intensive care units at Hamad Medical Corporation, Doha, Qatar, from November 2012 to October 2013.

**Methods:**

A total of 629 Enterobacteriaceae isolates were included in the study. Identification and susceptibility testing was performed using Phoenix (Becton Dickinson) and the ESBL producers were confirmed by double-disk potentiation as recommended by the Clinical and Laboratory Standards Institute. Molecular analysis of the ESBL producers was performed by polymerase chain reaction.

**Results:**

In total, 109 isolates (17.3 %) were confirmed as ESBL producers and all were sensitive to meropenem in routine susceptibility assays. Most of the ESBL producers (99.1 %) were resistant to amoxicillin/clavulanic acid and ceftriaxone and 93.6 % were resistant to cefepime. Among the ESBL-producing genes, *bla*_CTX-M_ (66.1 %) was the most prevalent, followed by *bla*_SHV_ (53.2 %) and *bla*_TEM_ (40.4 %).

**Conclusions:**

These findings show the high prevalence of ESBL-producing Enterobacteriaceae within the intensive care units at Hamad Medical Corporation, Qatar, and emphasize the need for judicious use of antibiotics and the implementation of strict infection control measures.

## Background

The emergence of extended-spectrum beta-lactamase (ESBL)-producing Enterobacteriaceae, particularly *Escherichia coli* and *Klebsiella pneumoniae*, presents a significant threat to human health and these organisms are now listed among the six drug-resistant pathogens for which there are few potentially effective drugs [1]. The first case of ESBL was reported in the 1980s in Europe and subsequently in the United States, soon after the introduction of third-generation cephalosporins [2]. Over the last two decades, there has been an exponential increase worldwide in β-lactamase production and the prevalence of ESBL-producing Enterobacteriaceae, contributing to a significant increase in antimicrobial resistance [3–5]. Importantly, infections with ESBL-producing pathogens are associated with poor clinical outcomes, longer hospital stays, higher mortality rates, and greater hospital expenses [6, 7]. In addition, there has been a rapid and widespread dissemination of ESBL-producing Enterobacteriaceae in communities as well as in hospital-associated infections, resulting in a worldwide health crisis [8].

The ESBL genes are predominantly plasmid encoded [9] and most belong to the class A β-lactamases, which can be divided into three genotypes: TEM, SHV, and CTX-M [6]. During the 1990s, ESBL-producing Enterobacteriaceae, *E. coli* and *K. pneumonia*, were described mainly as members of the TEM- and SHV-β-lactamase families [10]. Later in 2000, *K. pneumoniae* was reported as the major ESBL producer with TEM and SHV as the predominant genotypes [3]. However, ESBL-producing *E. coli* of the CTX-M genotype is now more prevalent in Western and Asian countries [3]. The CTX-M β-lactamases comprise more than 50 different types, which can be divided into five groups based on their amino acid identities: CTX-M-1, CTX-M-2, CTX-M-8, CTX-M-9, and CTX-M-25 [11, 12].

Several reports have described the prevalence of ESBLs in the Middle East North Africa (MENA) region and most of the Gulf Cooperation Countries [13]. However, there is insufficient scientific data on the epidemiology of ESBLs available from the State of Qatar. To the best of our knowledge, this is the first study on the molecular epidemiology and antimicrobial susceptibilities of ESBL-producing Enterobacteriaceae from patients in the State of Qatar.

## Methods

### Study design

The present prospective study was conducted on routine specimens received at the Microbiology Laboratory of the Department of Laboratory Medicine and Pathology, Hamad Medical Corporation (HMC), from patients admitted to the intensive care units (ICUs) at HMC. This study was approved (Protocol no. RC/75813/2013) by the Institutional Review Board of HMC.

### Clinical isolates/strains

A total of 629 Enterobacteriaceae isolates (a single isolate per patient) were collected between November 2012 and October 2013 from various clinical specimens as part of routine clinical care. These clinical isolates were preserved at −70 °C for further analysis. For each individual, age, nationality, and clinical history were collected from patients’ medical records. Infections occurring more than 48 h after admission were considered hospital acquired [14].

The standard strains of *E. coli* (ATCC 25922) and *K. pneumoniae* (ATCC 700603) were used for identification and antimicrobial drug susceptibility testing and *K. pneumoniae* (NCTC 13368), *E. coli* (NCTC 13351), and *E. coli* NCTC (13353) were used as positive controls for *bla*_SHV_, *bla*_TEM_, and *bla*_CTX-M_ in polymerase chain reaction (PCR) assays, respectively.

### Identification and antimicrobial drug susceptibility testing

The identification and antimicrobial susceptibility test was performed with Phoenix using the NMIC/ID-5 panel according to the manufacturer’s recommendations (BD Biosciences, Heidelberg, Germany). All samples, which tested positive for ESBL by Phoenix or showed an MIC of >2 μg/mL for ceftazidime, aztreonam, and ceftriaxone, were consequently confirmed by a double-disk potentiation test with ceftazidime, amoxicillin/clavulanic acid, ceftriaxone, and cefoxitin antibiotics and interpretation was carried out as previously described [15, 16]. Briefly, a microbial suspension with 0.5 McFarland turbidity was inoculated onto Mueller–Hinton agar (Oxoid Ltd., Basingstoke, Hampshire, England), followed by overnight incubation at 37 °C, and susceptibility/resistance patterns were determined.

### DNA extraction and detection of ESBL genes by PCR

Bacterial DNA was extracted using the boiling lysis method [17]. Briefly, a few colonies were suspended in Tris-EDTA buffer (pH 8.0). The suspensions were boiled at 100 °C for 10 min and subsequently centrifuged at 15871 g for 5 min. The supernatant was transferred to new tubes and stored at −20 °C for subsequent PCR analysis. PCR reactions were carried out using the following protocol: 2 μL of extracted DNA was combined with 12.5 μL of 2X master mix, 1 μL forward and 1 μL reverse primer, and the mixture was made up to a 25 μL volume with nuclease-free water. PCR amplification (Veriti 96 Well Thermal Cycler-Applied Biosystems, Pittsburg, PA, USA) was initiated at 96 °C for 1 min, followed by 35 cycles at 96 °C for 1 min, annealing (55 °C for TEM, and 50 °C for SHV and CTX-M-1) for 1 min, and extension at 72 °C for 2 min. Final extension was at 72 °C for 10 min. All samples were analyzed in duplicate. A positive control (plasmid carrying cloned ESBL gene fragment) and a negative control (nuclease-free water) were included in each amplification reaction. After the last cycle, the products were stored at 4 °C for further analysis. The PCR products (1/10 volume) were analyzed by gel electrophoresis (Bio-Rad, Hercules, CA, USA) using 2 % agarose gels in 1X TAE buffer (Tris-acetate EDTA). The gels were stained with ethidium bromide (Sigma, St. Louis, MO, USA), and the PCR products were visualized under ultraviolet light. A single band with an amplicon size of 1,150 bp was observed for TEM, 885 bp for SHV, and 499 bp for the CTX-M-1 group (Table 1) [18].

**Table 1:** Primers used for polymerase chain reaction amplification of extended-spectrum beta-lactamase genes (TEM, SHV and CTX-M-1)

Gene	Primer	Sequence (5′3′)	Amplicon size (bp)
TEM	TEM-F TEM-R	TTCTTGAAGACGAAAGGGC ACGCTCAGTGGAACGAAAAC	1,150
SHV	SHV-F SHV-R	CACTCAAGGATGTATTGTG TTAGCGTTGCCAGTGCTCG	885
CTX-M-1 group	CTXM1-F CTXM1-R	GACGATGTCACTGGCTGAGC AGCCGCCGACGCTAATACA	499

## Results

### Demographic characteristics of the study population

Samples were collected from patients with suspected bacterial infection from different ICUs (29.4 % from Medical, 28.5 % Surgical, 16.5 % Trauma, 15.6 % Pediatric, and 10 % from Neonatal) attached to HMC hospitals. Samples were subjected to culturing and subsequent pathogen identification. The demographic profile of the studied population is summarized in Table 2 and shows that 59.6 % (*n* = 65) of samples were collected from male patients and 40.4 % (*n* = 44) of samples were from female patients. Samples were collected from patients ranging in age from 1 month to 86 years, with a mean age of 40.31 years (SD = 26.79). It is noteworthy that 68.8 % of patients were non-Qatari and 86.2 % were admitted to hospital for >2 days.

**Table 2:** Demographic profile of the study population infected with extended-spectrum beta-lactamase-producing pathogens in the State of Qatar

	Total	Nationality		Hospital stay	
Gender ↓	No. (%)	Qatari	non-Qatari	<2 days	>2 days
Male	65 (59.6)	19	46	6	59
Female	44 (40.4)	15	29	9	35
Total No. (%)	109	34 (31.2)	75 (68.8)	15 (13.8)	94 (86.2)
Age groups (Years) ↓					
<1	18 (16.5)	5	13	4	14
1-12	8 (7.4)	4	4	2	6
13-30	13 (11.9)	2	11	0	13
31 -50	24 (22)	3	21	3	21
>50	46 (42.2)	20	26	6	40

### Distribution of clinical isolates

**Fig. 1:**
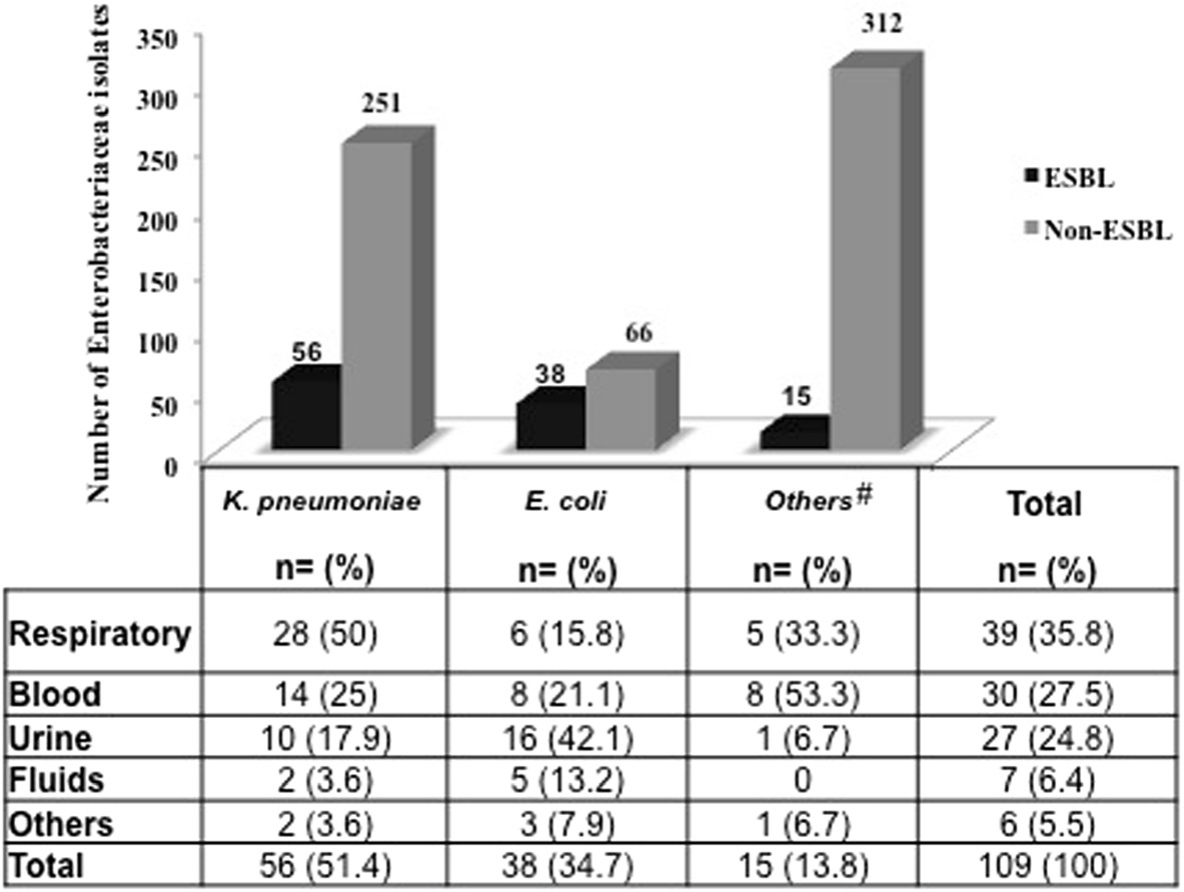
Distribution of extended-spectrum beta-lactamase-producing organisms among Enterobacteriaceae isolates and the site of isolation. # *Enterobacter cloacae, Citrobacter amalonaticus, Enterobacter aerogenes, Citrobacter amalonaticus, Serratia marcescens*, *Citrobacter braakii, Citrobacter freundii, Klebsiella oxytoca, Proteus penneri*

During the study period, a total of 109 (17.3 %) ESBL-producing Enterobacteriaceae were isolated. Amongst the isolates, *K. pneumonia*e (51.4 %, *n* = 56) was the most common ESBL-producing organism, followed by *E. coli* (34.7 %, *n* = 38), and the numbers of organisms other than *E. coli* and *K. pneumonia*e were low (13.8 %, *n* = 15) (Fig. 1). These pathogens were isolated from a variety of clinical samples, respiratory 35.8 % (*n* = 39), blood 27.5 % (*n* = 30), urine 24.8 % (*n* = 27), fluids 6.4 % (*n* = 7), and others 5.5 % (*n* = 6) (Fig. 1).

### Antimicrobial resistance/susceptibility profile of ESBL isolates

**Fig. 2:**
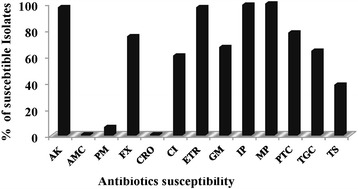
*In vitro* activity of the identification and antimicrobial susceptibility test panel of antimicrobial agents against clinical extended-spectrum beta-lactamase-producing isolates. The in vitro activity of antimicrobial agents against clinical isolates was analyzed by BD Phoenix. AK (amikacin), AMC (amoxicillin/clavulanic acid), PM (cefepime), FX (cefoxitin), CRO (ceftriaxone), CI (ciprofloxacin), ETR (ertapenem), GM (gentamicin), IP (imipenem), MP (meropenem), PTC (piperacillin/tazobactam), TGC (tigecycline), TS (trimethoprim/sulfamethoxazole)

Antibiotic resistance/susceptibility patterns of ESBL-producing pathogens are shown in Fig. 2. Among the carbapenems, all ESBL-producing isolates showed 100 % susceptibility to meropenem and slightly reduced susceptibility to imipenem (99.1 %) and ertapenem (97.2 %). Among the β-lactam/β-lactamase inhibitor combinations, 78 % were sensitive to piperacillin/tazobactam, whereas 99.1 % were resistant to amoxicillin/clavulanic acid. Susceptibilities to cephalosporins were found to be very low (ceftriaxone 0.9 % and cefepime 6.4 %). The clinical isolates showed high sensitivity to amikacin (97.2 %) compared with gentamicin (67 %) amongst the aminoglycosides. The susceptibility to other classes of antimicrobials such as tigecycline, ciprofloxacin, and trimethoprim/sulfamethoxazole was relatively low (64.2 %, 60.6 %, and 38.5 % respectively).

### Molecular genotyping of ESBL-producing isolates

**Fig. 3:**
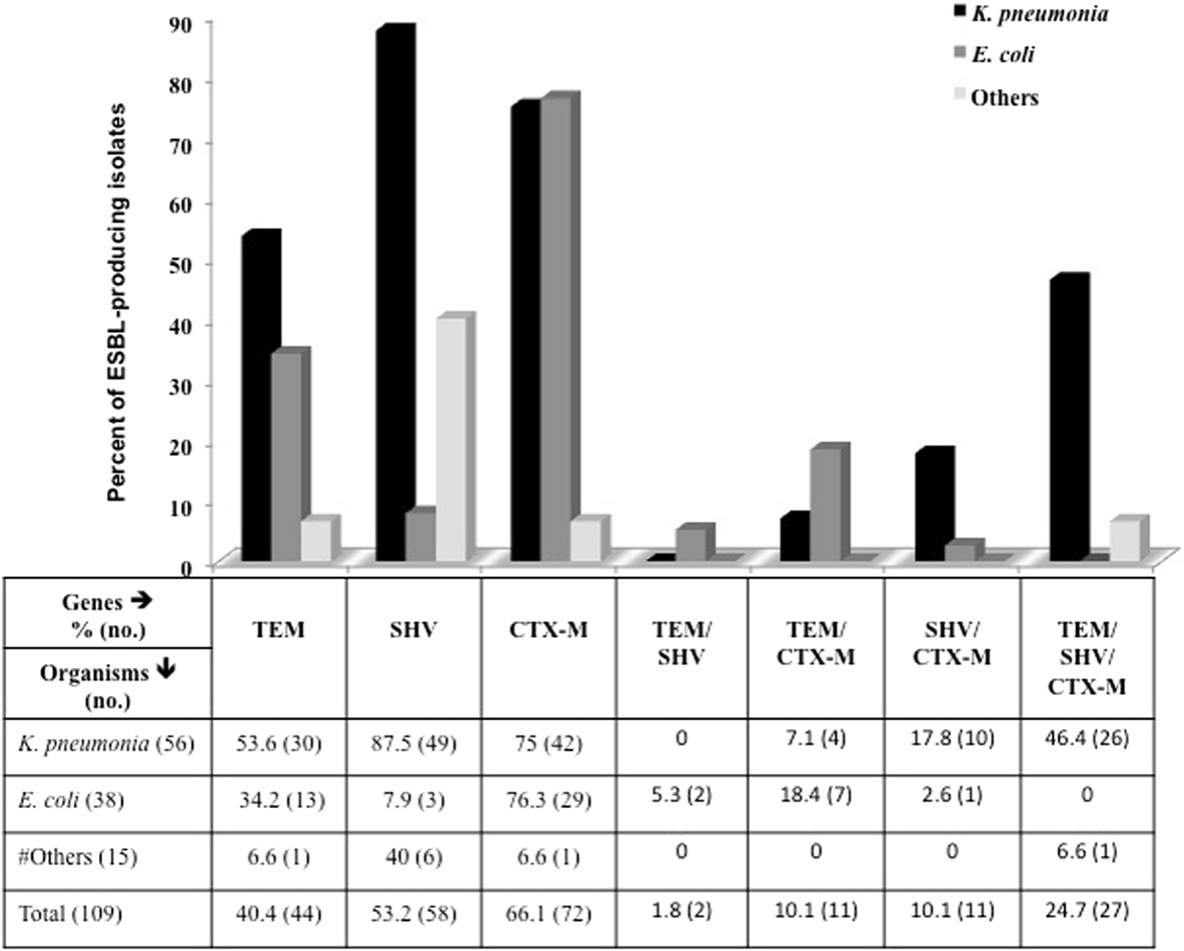
Distribution of extended-spectrum beta-lactamase-producing genes (TEM, SHV and CTX-M-1) among Enterobacteriaceae isolates in the State of Qatar. # *Enterobacter cloacae, Citrobacter amalonaticus, Enterobacter aerogenes, Citrobacter amalonaticus, Serratia marcescens*, *Citrobacter braakii, Citrobacter freundii, Klebsiella oxytoca, Proteus penneri*

The ESBL-producing pathogens confirmed by phenotypic methods were also molecularly analyzed. Of the 109 ESBL isolates, 24.7 % harbored multiple *bla* genes simultaneously and the prevalence of *bla*_CTX-M_ was highest at 66.1 %, followed by *bla*_SHV_ at 53.2 %, and *bla*_TEM_ at 40.4 % (Fig. 3). The majority of the CTX-M-1-positive isolates were *E. coli* (76.3 %) and *K. pneumoniae (*75 %); however, all TEM and SHV-positive isolates were *K. pneumoniae* (53.6 % and 87.5 % respectively). Furthermore, all three *bla* genes (TEM, SHV, and CTX-M-1) were detected in 46.4 % of *K. pneumonia*e isolates*,* while two genes (SHV/CTX-M-1) were present in 17.8 % of *K. pneumonia*e and 2.6 % of *E. coli* isolates, with TEM/CTX-M-1 being present in 18.4 % of *E. coli* and 7.1 % of *K. pneumoniae* and TEM/SHV being detected in only 5.3 % of *E. coli* isolates (Fig. 3). None of the *bla* genes were detected in *Serratia marcescens*, *Citrobacter braakii*, *Citrobacter freundii*, *Klebsiella oxytoca*, or *Proteus penneri* (data not shown).

## Discussion

In this study, we examined the prevalence of ESBL-producing Enterobacteriaceae and carried out molecular characterization and antimicrobial susceptibility testing of clinical samples from patients admitted to ICUs at HMC, Doha, Qatar. A total of 629 Enterobacteriaceae isolates were evaluated for the production of ESBL enzymes. In our study, the major ESBL producer was found to be *K. pneumoniae* followed by *E. coli* and other species, which is in agreement with previous studies [13, 19], but overall the prevalence of ESBL-producing pathogens was substantially lower (17.3 %) than that reported by other studies from neighboring MENA and Asian countries [13, 20–26]. Methodological differences may explain the differences observed in the measured prevalence levels.

In the present study, the respiratory tract was the major source of ESBL-producing isolates, followed by the blood and other sampling sites. Similar findings have been reported for the ICUs of tertiary care hospitals in Mexico and India, where the major source of ESBL-producing isolates were the respiratory tract and blood, respectively [27, 28]. However, in the Gulf Cooperation Countries region, urine and blood were reported as the major source of ESBL-producing bacteria [13, 26, 29].

The highest level of resistance, in the current study, was observed to be amoxicillin/clavulanic acid, ceftriaxone, and cefepime, while all isolates were susceptible to meropenem, with decreasing levels of susceptibility to imipenem, ertapenem, amikacin, piperacillin/tazobactam, gentamicin, tigecycline, ciprofloxacin, and trimethoprim/sulfamethoxazole. Recently, Somily et al. [30] reported similar susceptibility rates among *E. coli* and *K. pneumonia*e isolates from a tertiary care hospital at Riyadh, Kingdom of Saudi Arabia. However, susceptibility to piperacillin/tazobactam and ciprofloxacin were lower in our study compared with isolates from a hospital at Dammam, Kingdom of Saudi Arabia [26]. Furthermore, in Sudan, ESBL-producing *E. coli* were highly resistant to trimethoprim/sulfamethoxazole and ciprofloxacin but less resistant to amoxicillin/clavulanic acid compared with the present study, but similar susceptibility rates were observed to amikacin and gentamicin [29]. The resistance observed with ertapenem and imipenem compared with merepenem could possibly result from carbapenemase production and/or resistance owing to the loss of porins and/or hyper-production of AmpC; however, this was not further evaluated because of the limitations of the present study.

The predominant genotype of ESBL-producing *E. coli* and *K. pneumonia* has changed from TEM and/or SHV to CTX-M-1 [3], and currently, CTX-M has been reported to be the most prevalent genotype among ESBL-producing isolates [13, 26, 28, 31–33]. In this study, molecular genotyping of ESBL-positive isolates showed that the CTX-M-1 gene was the most common among *E. coli* and *K. pneumonia* followed by SHV and TEM, which was consistent with previous reports from Turkey and India [34, 35]. Additionally, our results showed that only 24.7 % of isolates produced all three genes concurrently and 10.1 % of isolates co-produced TEM/CTX-M-1 and SHV/CTX-M-1. These findings obscure the detection rates, affect subsequent treatment strategies, and could be the reason for resistance to β-lactamase inhibitors [26].

## Conclusions

Compared with other countries in the MENA region, our study shows relatively low prevalence (17.3 %) of ESBL-producing Enterobacteriaceae. Though lower than in other countries and regions, our study suggests that there is sufficient infection burden to warrant public health interventions. Notably, the majority of isolates were multi-drug resistant and belonged to plasmid-type CTX-M. The emergence of CTX-M-producing Enterobacteriaceae isolates is of major concern and highlights the need for further surveillance in this area. As meropenem shows good activity against these ESBL producers, it should be restricted for managing patients with suspected Gram-negative bacterial infections with ESBL production. Additionally, antimicrobial stewardship and early detection by active surveillance coupled with an effective infection control program are key to reducing or halting the spread of ESBL producers in hospitals and communities in the State of Qatar.

### Compliance with ethical guidelines

#### Ethical standard

This study was approved by the Research Ethics Committee at HMC, Doha, Qatar.
